# Does integration of HIV and SRH services achieve economies of scale and scope in practice? A cost function analysis of the Integra Initiative

**DOI:** 10.1136/sextrans-2015-052039

**Published:** 2015-10-05

**Authors:** Carol Dayo Obure, Lorna Guinness, Sedona Sweeney, Integra Initiative, Anna Vassall

**Affiliations:** Faculty of Public Health and Policy, Department of Global Health and Development, London School of Hygiene and Tropical Medicine, London, UK

**Keywords:** HIV, REPRODUCTIVE HEALTH, ECONOMIC ANALYSIS, AFRICA

## Abstract

**Objective:**

Policy-makers have long argued about the potential efficiency gains and cost savings from integrating HIV and sexual reproductive health (SRH) services, particularly in resource-constrained settings with generalised HIV epidemics. However, until now, little empirical evidence exists on whether the hypothesised efficiency gains associated with such integration can be achieved in practice.

**Methods:**

We estimated a quadratic cost function using data obtained from 40 health facilities, over a 2-year-period, in Kenya and Swaziland. The quadratic specification enables us to determine the existence of economies of scale and scope.

**Findings:**

The empirical results reveal that at the current output levels, only HIV counselling and testing services are characterised by service-specific economies of scale. However, no overall economies of scale exist as all outputs are increased. The results also indicate cost complementarities between cervical cancer screening and HIV care; post-natal care and HIV care and family planning and sexually transmitted infection treatment combinations only.

**Conclusions:**

The results from this analysis reveal that contrary to expectation, efficiency gains from the integration of HIV and SRH services, if any, are likely to be modest. Efficiency gains are likely to be most achievable in settings that are currently delivering HIV and SRH services at a low scale with high levels of fixed costs. The presence of cost complementarities for only three service combinations implies that careful consideration of setting-specific clinical practices and the extent to which they can be combined should be made when deciding which services to integrate.

**Trial registration number:**

NCT01694862.

## Introduction

Policy-makers and researchers have long argued about the potential benefits of integrating HIV prevention, treatment and care with sexual and reproductive health (SRH) services in settings with generalised HIV epidemics. Since the global economic crisis, interest in the potential efficiency gains from service integration has increased, as the ability of developed countries to fulfil commitments to fund the full coverage of HIV programmes in developing countries has been questioned.^1^

The notion that efficiencies may be gained through integration of HIV and SRH services has considerable intuitive and theoretical appeal. Efficiency gains or cost savings may be achieved through economies of scale and scope. Economies of scale are defined as cost savings resulting from an increase in scale of operations. These may occur when integration results in an increase in demand and provision of services, hence lowering the unit cost of production. On the other hand, economies of scope measure cost savings that occur from producing several outputs simultaneously rather than separately. Economies of scope can arise from (1) cost complementarity between two outputs or (2) spreading fixed costs over an expanded output mix. Cost complementarities occur when adding a new service reduces the marginal or average incremental cost of delivering another service. Spreading fixed costs contributes to economies of scope when excess capital capacity is reduced by producing HIV and SRH services together rather than separately.

Despite the theoretical and policy importance of this question, very few studies have empirically evaluated the efficiency gains associated with integrated delivery of HIV and SRH services. While there is a growing body of evidence on the social, behavioural and health benefits of integrating HIV services into SRH services,^2–4^ reviews have consistently noted a substantial dearth of evidence on the cost savings and improved efficiency of delivering integrated HIV and SRH services.^3–7^ The few studies until now suggesting that integration of HIV services into SRH services yields cost savings were either conducted at a relatively small scale or with study designs that were unable to establish statistically significant results.^8–12^

The objective of this study is therefore to estimate a multi-output cost function for integrated HIV and SRH service delivery to evaluate the existence of economies of scale and scope in a sample of health facilities in Kenya and Swaziland.

## Methods

### Theoretical framework

To evaluate the existence of economies of scale and scope, we estimated a hybrid cost function.^13–15^ The hybrid cost function combines output volumes, input prices and organisational variables to explain total costs of HIV/SRH services. We estimated a quadratic cost function and specify the cost function as a random effects generalised least squares (GLS) model with u_i∼iid_ (o, 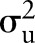). The quadratic functional form is chosen because unlike the trans-logarithm functional form, it accommodates zero values for outputs therefore allowing for straightforward identification of economies of scope. The functional form is written as
1
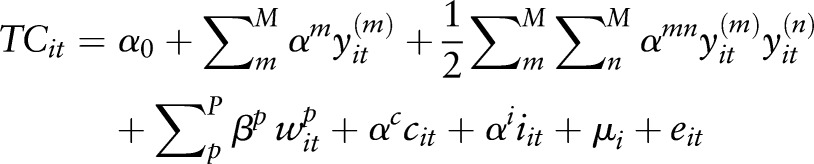

where TC_it_ represents the total costs for HIV and SRH services. The superscripts m and p denote the number of outputs and the number of input factors and subscripts i and t denote the health facility and the year. y_it_ refers to outputs (in our case m,n=6 HIV/SRH outputs). We include linear terms for input prices (w), the proportion of clinical staff (c), measures of integration (i) and quadratic terms for outputs as well as interaction terms with the outputs. The terms µ_i_ and e_it_ represent the firm-specific individual effects and the error term. Finally, α_0_ is the constant.

### Economies of scale and scope

Two distinct concepts of economies of scale apply in a multi-output (service) setting: service-specific economies of scale and ray (overall) economies of scale. Service-specific economies of scale (EOS_1_) occur when increases in service y^1^ result in declining average incremental costs. For example, as the level of a specific HIV or SRH services increases, the cost of providing additional services declines. The degree of service-specific economies of scale is given by
2
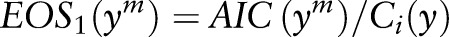

where AIC (y^m^) is the average incremental cost of the mth service. In this case, if EOS_1_ (y^m^) is greater (less) than 1, then economies (diseconomies) of scale are said to exist for the mth service.

Ray economies of scale (RES) describe the behaviour of costs as all outputs are increased by the same proportion. Following Baumol *et al*,^16^ the degree of RES in a multi-output setting is defined as
3
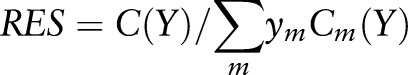

where C(Y) is the total cost of producing all n outputs (Y_m)_) and Ci=δC(Y)/δY_m_ is the marginal cost of producing the mth output.

RES are said to exist if RES >1 and ray diseconomies of scale are said to exist if RES <1.

The conventional measure of the degree of economies of scope is defined as the ratio of excess costs of separate production to the costs of joint production of all outputs. Therefore, economies of scope exist when the cost of joint production is less than the sum of the costs of separately produced outputs or subset of outputs. However, following Baumol *et al*,^16^ when there are zero values for some output types, as is the case in this study, weak cost complementarities (WCC) can be used as a sufficient condition for economies of scope. Under this empirical test, we investigate how an increase in one of the six services affects the marginal cost of producing the other services. WCC occurs when increases in one output reduce the marginal cost of other outputs. This occurs if the expression:
4
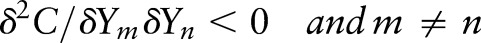


Equation (4) states that cost complementarities between two outputs are present when the marginal cost of producing one output decreases as the quantity of the other output increases.

### Data

This paper used data collected as part of a large non-randomised trial, Integra Initiative (Clinical Trials.gov identifier: NCT01694862) designed to evaluate the costs and benefits of integrating HIV and SRH services in Kenya, Swaziland and Malawi.^17^

The Integra Initiative was originally designed as a pre-study/post-study with pair-matched intervention (integrated) and comparison (non-integrated) sites. However, with external donor activities and evolving national policy the control sites also integrated services during the study period resulting in no distinguishable control and intervention sites.

The data used for our empirical analysis contain cost, HIV and SRH service utilisation information and health facility characteristics obtained from 40 health facilities observed over a 2-year-period 2008–2009 and 2010–2011 (n=80). The study sample included 30 health facilities in Kenya and 10 in Swaziland. Of the 40 health facilities, 80% were public health facilities, 43% were located in urban areas and 26% were classified as hospitals with inpatient facilities. The sample included a range of facility types, including hospitals, health centres, public health units and SRH clinics.

Written informed consents were obtained for all Integra Initiative activities.

The variables used in the cost function were constructed as follows. Total costs (TC_it_) were calculated as the total economic costs of service delivery at the health facilities for a given year. To obtain estimates of total economic costs of HIV/SRH services, costs were initially classified into two main categories: capital and recurrent costs. Capital costs included buildings, equipment and training costs. All capital costs were annualised and discounted at the standard rate of 3%.^18^ Recurrent costs included staff salaries, building maintenance (including utility expenses), drugs, medical and non-medical supplies, transport and diagnostics. All costs of overhead/administrative and support departments (laboratory/ pharmacy) were allocated to the HIV/SRH services using the top-down costing approach. The top-down costing approach identifies the total resources required to deliver services and then assigns these resources to specific activities or services based on allocation criteria such as floor space, personnel hours or activity data.^19^ Further details of the costing methods used are reported in detail elsewhere.^20^

We define six outputs (y^n^) measured as the number of visits for SRH and HIV services. SRH services included family planning (FP), post-natal care (PNC) and cervical cancer (Ca Cx) screening. HIV services included counselling and testing for HIV (HCT), treatment of sexually transmitted infections (STI) and HIV treatment and care (HIV). Data on the total number of visits were collected from registers and monthly reports.

Input prices were defined as average annual wages for clinical and technical staff, and computed as total annual wages for clinical and technical staff divided by the corresponding number of staff. Information on staff wages was obtained from the Ministries of Health (MoHs) for the public health facilities and the non-governmental organisation (NGO) headquarters for the NGO facilities. Although wages for the public health facilities did not differ within each employee category, health facilities exhibited heterogeneous staff mixes and therefore average wages varied across facilities. Prices of capital stock and equipment were not included as these were valued using standard national prices and therefore did not vary across health facilities studied.

As covariates, we considered the ratio of clinical staff to total staff as a proxy for the quality of labour complement at each health facility. We also explored differences in the extent of integration using a number of measures. First, we used the standard measure of integration—the range of HIV/SRH services provided within the facility and the range of HIV/SRH services provided within the maternal and child health (MCH) unit. To capture the extent of physical integration, we also included a variable on the range of HIV/SRH services provided per clinical room in the MCH unit and the range of HIV/SRH services provided per clinical staff member per day.

Second, an index of functional integration describing service utilisation patterns from the client perspective was also used. The index summarised four characteristics of service delivery: the extent to which HIV treatment was reported as being offered on site (or referred for); the range of services reported as received during the week; the range of services reported as received in a single consultation and the range of services reported as received in single visits. The functional integration index was developed using latent variable techniques with data obtained from ‘client flow’ surveys. These measures of integration were constructed using data collected from health facility registers as well as a client flow analysis and a health facility assessment carried out as part of the larger Integra Initiative.^17^ Further details on the creation of this index are provided in Mayhew *et al* (S Mayhew, GB Ploubidis, K Church, *et al*. Innovation in the evaluation of service Integration: the Integra Indexes of HIV and Reproductive Health Integration. Unpublished work, 2014).

We estimate two specifications of the cost functions using the measures of integration. The first specification includes the individual measures of integration as covariates, while the second includes the functional index of integration.

Table 1 provides a descriptive summary of the variables included in the cost function. All costs and prices are adjusted to 2014 US$.

**Table 1:** Descriptive statistics of variables used in the empirical study

Variable	Variable description	Mean	SD	Min.	Max.
Total cost (TC)	Total annual HIV and SRH costs (US$ 2014)	258 898.80	501 791.10	2513	2 703 186
y^1^	Total family planning visits	3887	3592	470	22 094
Y^2^	Total post-natal care visits	687	867	0	3330
Y^3^	Total cervical cancer screening visits	203	362	0	2063
Y^4^	Total HIV counselling and testing visits	2670	2851	0	15 878
Y^5^	Total STI treatment visits	277	667	0	3702
Y^6^	Total HIV treatment visits	3747	9917	0	70 605
Pc	Average annual wage per clinical staff	9059.68	6480.74	1427.28	37 552.96
Pt	Average annual wage per technical staff	3145.69	2675.92	0	11 102.34
I^1^	Range of HIV/SRH services provided in the facility	6.64	1.09	3	8
I^2^	Range of HIV/SRH services provided in the MCH unit	2.26	1.14	0	4
I^3^	Range HIV/SRH service provided per clinical staff per day	1.92	0.97	0	4
I^4^	Range HIV/STI services provided per room per day	1.26	0.88	0	4
I^5^	Functional integration index score	0.01	0.94	−1.25	3.59
Cs	Proportion of clinical staff	0.49	0.15	0.17	0.95

SRH, sexual reproductive health; STI, sexually transmitted infections.

## Results

The empirical results of the two specifications of equation (1) estimated using the quadratic functional form are presented in table 2. The results of the Hausman specification test reveal that the coefficients estimated by the chosen random effects estimator are not statistically different from those obtained by the fixed effects model. This inspires confidence that the model is correctly specified.

**Table 2:** Regression results of the GLS model

Dependent variable= (total annual cost)	Specification 1		Specification 2	
	Coeff.	SE	Coeff.	SE
Y^1^	48.02**	19.972	40.883*	21.803
Y^1^Y^1^	−0.0061	0.0001	0.00020	0.0011
Y^2^	13.20	89.551	−12.99976	95.088
Y^2^Y^2^	−0.0279	0.0339	−0.019	0.036
Y^3^	539.77***	201.760	−188.9234	197.664
Y^3^Y^3^	0.2227**	0.0974	0.116	0.103
Y^4^	−49.747**	22.231	−10.55806	23.459
Y^4^Y^4^	0.0012	0.0016	0.001	0.001
Y^5^	−460.208***	138.453	−212.193	144.959
Y^5^Y^5^	0.502***	0.071	0.429***	0.073
Y^6^	25.617**	10.241	35.556***	9.224
Y^6^Y^6^	0.0026***	0.0006	0.002***	0.0007
Y^1^Y^4^	0.0256	0.005	−0.001	0.005
Y^1^Y^5^	−0.1000***	0.020	−0.103***	0.020
Y^1^Y^6^	−0.0017	0.0015	−0.003**	0.001
Y^2^Y^4^	0.031**	0.073	0.017	0.015
Y^2^Y^5^	−0.062*	0.035	−0.010	0.038
Y^2^Y^6^	−0.033***	0.008	−0.026**	0.009
Y^3^Y^4^	0.023	0.0211	0.001	0.023
Y^3^Y^5^	−0.575***	0.170	0.280	0.184
Y^3^Y^6^	−0.132***	0.034	−0.096***	0.037
^Pc^	16.22***	5.003	24.882***	5.359
P^t^	−20.47**	8.575	−13.322	9.457
I^1^	20 610.82	26 131.61	–	–
I^2^	159 054.8***	40 892.33	–	–
I^3^	−180 977.2***	40 672.14	–	–
I^4^	26 846.94	32 925.64	–	–
I^5^	–	–	773.3802	20 381.52
Cs	−60 292	141 372.9	−117 282.9	159 463.1
Intercept	−94 982.13	121 136.5	−41 241.97	100 017.1
N	80		80	

***p<0.01, **p<0.05, *p<0.10.

Y^1^, family planning visits; Y^2^, post-natal care visits; Y3, Ca Cx visits; Y^4^, HCT visits; Y^5^, sexually transmitted infection visits; Y^5^, HIV visits.

These results show that only the output coefficients for FP and HIV outputs are significant in both specifications. While the coefficients for input prices are both significant for the first specification, only the price coefficient for clinical staff is significant for the second specification. In both specifications, only the FP and HIV output coefficients and the clinical staff input price coefficient have the expected positive sign.

As expected, the effect of range of services per clinical staff (coefficient I^3^) was negative and significant, which indicates that an increase in the range of services per clinical staff decreases costs significantly.

Another interesting observation is the positive but not significant coefficient for functional integration. This suggests that functional integration does not have a significant effect on costs.

### Economies of scale and scope

Table 3 presents the estimated values of economies of scale and scope. These were estimated using the cost equation (1) and the formulas specified in equations (3) and (4) for each of the health facilities included in the sample. The results suggest that service-specific economies of scale exist for only HIV counselling and testing (HCT) services. However, the estimates of RES for both specifications were <1, suggesting that overall economies of scale do not exist for the HIV and SRH services. These results further suggest that at their current output levels, the health facilities included in the sample are not fully exploiting their potential economies of scale.

**Table 3:** Estimates of economies of scale and weak cost complementarities (WCC)

Service-specific economies of scale	
Y1 (FP)	0.005
Y2 (PNC)	0.001
Y3 (Ca Cx screening)	0.002
Y4 (HCT)	1.002
Y5 (STI treatment)	0.039
Y6 (HIV care)	0.158
Ray economies of scale	−0.001
WCC	
Y^1^ (FP)×Y^4^ (HCT)	0.03
Y^2^ (PNC)×Y^4^ (HCT)	0.03**
Y^3^ (Ca Cx screening)×Y^4^ (HCT)	0.02
Y^1^ (FP)×Y^5^ (STI treatment)	−0.10***
Y^2^ (PNC)×Y^5^ (STI treatment)	−0.06*
Y^3^ (Ca Cx screening)×Y^5^ (STI treatment)	−0.57***
Y^1^ (FP)×Y^6^ (HIV care)	−0.00
Y^2^ (PNC)×Y^6^ (HIV care)	−0.03**
Y^3^ (Ca Cx screening)×Y^6^ (HIV care)	−0.13***

***, ** and * denote significance at the 1%, 5% and 10% levels, respectively.

FP, family planning; PNC, post-natal care; STI, sexually transmitted infections.

We also note significantly negative interaction coefficients for Y^1^Y^5^ (FP and STI treatment); Y^2^Y^6^ (PNC and HIV care) and Y^3^Y^6^ (Ca Cx screening and HIV care in both specifications suggesting cost advantages from jointly providing these outputs). The positive interaction term for Y^1^Y^4^ (FP and HCT), although not significant, suggests no cost advantages from jointly producing these two outputs, which is puzzling.

## Discussion

Overall we found that integration, measured using conventional measures of integration (such as the range of services provided in either the facility or the MCH unit), may increase the total cost of service delivery. However, while a more integrated service mix as measured by the functional integration has little impact on total costs, an increase in the range of services provided by clinical staff can reduce total costs of service delivery. The disparity in these findings probably reflects the fact that studies such as these are unable to account for case mix or quality—and it is conceivable that integration may improve both, but at additional cost. The increased total cost from the range of services may, for example, reflect a more comprehensive, higher quality service offered for FP clients.

The findings on the impact of integration on cost are further refined through our examination of economies of scale and scope—which provides additional insight into which combinations of SRH/HIV services are likely to achieve the most cost savings. We found evidence of service-specific economies of scale for STI and HCT services, in line with other studies,^21–23^ but no evidence of global economies of scale in integrated SRH/HIV services—as all services are expanded. This may reflect the extent to which these particular services rely on ‘fixed’ staff and capital costs, rather the relatively high variable costs common in HIV care and treatment.

The positive significant coefficients on the scope effect for FP and HCT services suggest that these services could be provided independently without significant negative effects on costs. While it has been argued that there is a low marginal cost to providing HCT during a FP visit,^24^ FP and HCT services in practice may have different patterns of resource requirements. Counselling for HIV may be provided together with FP counselling with no additional resource requirement; however, testing for HIV requires not only additional staff time but also equipment and supplies to process the test results, and therefore in practice there may be limited savings from these specific services being jointly provided.

Some limitations of this study should be noted. First, although this is one of the largest studies until now on the impacts of integrating HIV and SRH services in a low-income and middle-income setting, the results obtained from this study lack the statistical power of larger panel data sets. This limits the strength of the conclusions that can be drawn. Also, although the study captures the heterogeneity in health facilities in terms of HIV and SRH services provided, no case mix variables were able to be included to control for complexity of services provided because of unavailability of such data in the study setting. Finally, as with all studies of this kind, we cannot establish causality, due to the lack of an experimental design. However, the approach we adopt is commonly accepted as a sound basis for exploring associations. There are substantial challenges in controlling for the level of integration in real-world settings, meaning that few have succeeded until now to provide experimental evidence in this respect.

Despite these limitations, our findings have implications for both the planning for and the organisation of HIV and SRH services at the facility level generally. First, while the intuitive case for integration remains strong, in practice only some forms of integration may have this consequence. The results of this study have shown that efficiency gains from joint production are dependent on the specific combination of resources used for different services. These may vary by setting but, in general, this study adds to the evidence that HIV and SRH services that have substantial fixed costs are most likely to exhibit economies of scale. The specific costs that are fixed will, however, vary by setting. For example, in rural settings, staffing complements may be fixed; and therefore there may be substantial gains by increasing the volume of services provided by this minimum staffing complement, through increasing the range of services offered. With respect to economies of scope, this study suggests that planners in all settings need to carefully consider the detailed processes and clinical practice required by each service, and which of these can be combined when services are integrated, before assuming substantial cost advantages. The extent to which the specific findings of this study can be directly generalised depends on how similar the process of service delivery and clinical practice are to those observed here. Finally, it should be noted that integration has many aims (and consequences) and that the considerations above are only one factor in the optimal service design. As such, careful consideration should be made to optimally balance cost with other service delivery aims.

## Conclusion

This paper sets out to evaluate the existence of economies of scale and scope. We estimated a quadratic cost function using data obtained from 40 health facilities providing integrated HIV and SRH services in Kenya and Swaziland. The results from this analysis reveal that contrary to expectation, efficiency gains that can be reasonably expected from integration of HIV and SRH services, if any, are likely to be low.

**Key messages**

- Efficiency gains that can be reasonably expected from integration of HIV and sexual reproductive health services, if any, are likely to be modest.
- No evidence of overall economies of scale suggesting that health facilities included in the sample are not exploiting potential economies of scale.
- Cost complementarities were found for cervical cancer screening and HIV treatment; family planning and sexually transmitted infection treatment and post-natal care and HIV treatment only.
- The extent to which the specific findings of this study can be directly generalised depends on how similar the process of service delivery and clinical practice are to those observed here.
